# Endoscopic Complications Are More Frequent in Levodopa–Carbidopa Intestinal Gel Treatment via JET-PEG in Parkinson’s Disease Patients Compared to Nutritional PEG in Non-Parkinson’s Disease Patients

**DOI:** 10.3390/jcm13030703

**Published:** 2024-01-25

**Authors:** Laura Gombošová, Jana Deptová, Ivana Jochmanová, Tatiana Svoreňová, Eduard Veseliny, Mária Zakuciová, Vladimír Haň, Alexandra Lacková, Kristína Kulcsárová, Miriama Ostrožovičová, Joaquim Ribeiro Ventosa, Lenka Trcková, Ivica Lazúrová, Matej Škorvánek

**Affiliations:** 1 2nd Department of Internal Medicine, Faculty of Medicine, University Hospital of Louis Pasteur, Pavol Jozef Šafárik University, 04001 Košice, Slovakia; eduard.veseliny@upjs.sk (E.V.); maria.zakuciova@unlp.sk (M.Z.); 2 1st Department of Internal Medicine, Faculty of Medicine, University Hospital of Louis Pasteur, Pavol Jozef Šafárik University, 04001 Košice, Slovakia; ivana.jochmanova@upjs.sk (I.J.); ivica.lazurova@upjs.sk (I.L.); 3 Department of Neurology, Faculty of Medicine, Pavol Jozef Šafárik University, 04001 Košice, Slovakia; tatiana.svorenova@upjs.sk (T.S.); vladimir.han@upjs.sk (V.H.); alexandra.lackova@unlp.sk (A.L.); kristina.kulcsarova@upjs.sk (K.K.); miriama.ostrozovicova@unlp.sk (M.O.); joaquim.ribeiro.ventosa@upjs.sk (J.R.V.); matej.skorvanek@upjs.sk (M.Š.); 4 Department of Neurology, University Hospital of Louis Pasteur, 04001 Košice, Slovakia; lenka.trckova@unlp.sk; 5 Department of Clinical Neurosciences, University Scientific Park MEDIPARK, Pavol Jozef Šafárik University, 04001 Košice, Slovakia

**Keywords:** percutaneous endoscopic gastrostomy (PEG), levodopa–carbidopa intestinal gel therapy, advanced Parkinson’s disease, complications

## Abstract

**Background**: To date, no studies comparing complication rates between patients with nutritional percutaneous endoscopic gastrostomy (N-PEG) and Parkinson’s disease (PD) patients with percutaneous endoscopic gastro-jejunostomy (JET-PEG) for treatment administration have been published. Our study aimed to compare complication rates and the number of re-endoscopies between N-PEG and JET-PEG patients. **Methods**: Individuals requiring N-PEG or JET-PEG insertion between 2014 and 2021 were included in this single-center retrospective observational study. Complications were divided into time-related medical and technical complications. Reasons for post-insertion re-endoscopies and their number were also analyzed. **Results**: Eighty-seven subjects, 47 (54.02%) in JET-PEG group and 40 (45.98%) in the N-PEG group, were included. Early and technical complications were more frequent in JET-PEG vs. N-PEG subjects (70% vs. 10% [*p* < 0.001], and 54.5% vs. 5.1% [*p* < 0.001], respectively). The presence of psychiatric disease was associated with a higher number of early complications (*p* < 0.002). All three types of complications were significantly more frequent in subjects where a healthcare professional did not handle PEG (*p* < 0.001). Subjects with JET-PEG required a higher number of re-endoscopies compared to the N-PEG group (57.1% vs. 35%, *p* = 0.05). **Conclusions**: Complications are significantly more common in individuals with JET-PEG than those with N-PEG, which can be attributed to higher mobility in PD patients.

## 1. Introduction

Percutaneous endoscopic gastrostomy (PEG) is a widely used method of enteral feeding in patients with impaired ingestion but a normal function of the gastrointestinal (GI) tract and stomach [1,2]. In addition to nutritional PEG (N-PEG), percutaneous endoscopic gastrojejunostomy (JET-PEG, JET – jejunal extension tube) is used in device-aided therapy of subjects with advanced Parkinson’s disease (PD) and troublesome motor fluctuations with insufficient control on oral medication. This approach is based on the continuous delivery of levodopa–carbidopa intestinal gel (LCIG) via a portable pump directly to the proximal small intestine to achieve stable plasmatic levels of levodopa and a sustained control of the PD motor symptoms throughout the day [3]. This system administers the drug directly to the site of absorption, bypassing the stomach, which minimizes problems associated with delayed or variable gastric emptying or other peripheral pharmacokinetic issues [4].

Several studies have reported on the rates of complications for N-PEG and JET-PEG procedures separately; however, there are no previous studies directly comparing these methods. Importantly, while the procedure of PEG insertion is technically similar, a major difference is the targeted patient population. Compared to the typical patients who are candidates for N-PEG placement, the LCIG-treated PD patients are all conscious, mobile, and often presenting with motor fluctuations, in some cases including bothersome hyperkinetic movements (dyskinesia) [5]. Despite N-PEG patients generally suffering from more severe multi-morbidity and malnutrition, the available literature and experience consistently show that JET-PEG LCIG-treated PD subjects are more likely to encounter both early and late complications after PEG insertions; nevertheless, the factors driving this difference have not been clearly elucidated [6,7]. The aim of our study was, thus, to assess and compare the frequency and potential determinants of early- and late-onset medical and technical complications between N-PEG and JET-PEG groups and discuss their management. To date, no studies directly comparing complication rates in patients with N-PEG or Parkinson’s disease patients treated with LCIG through JET-PEG have been published.

## 2. Material and Methods

### 2.1. Study Design

**Figure 1:**
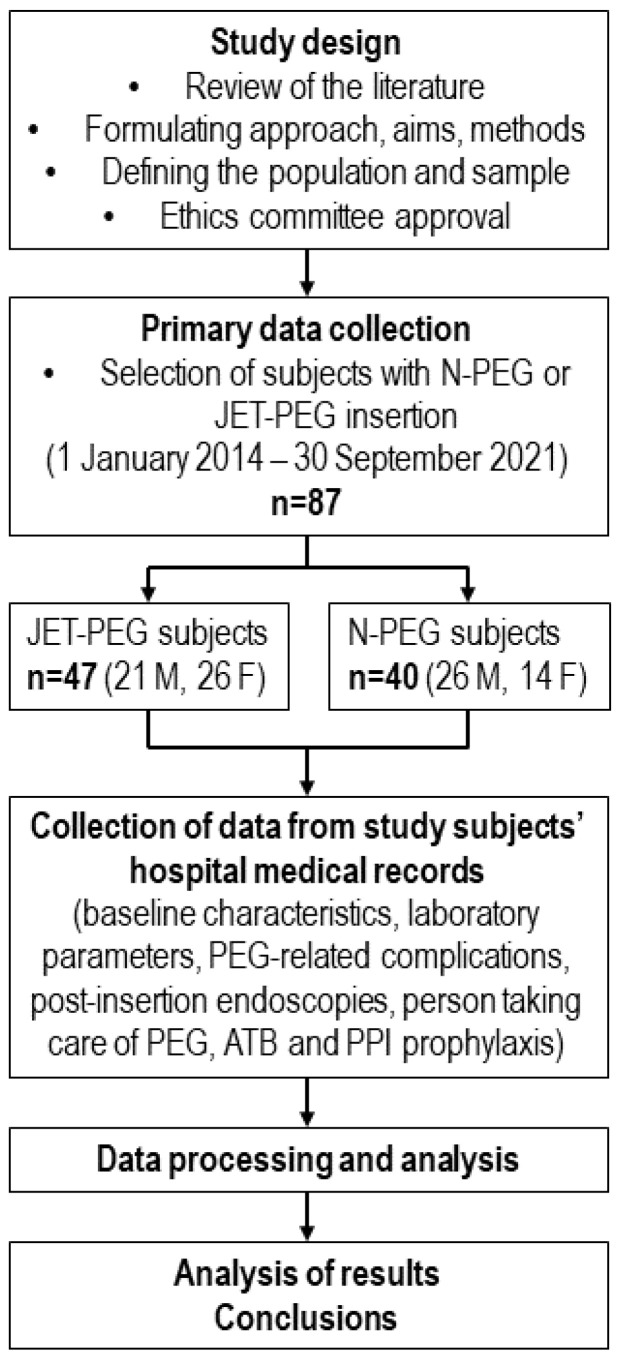
The study flow diagram. Abbreviations: ATB—antibiotics; F—females; JET-PEG—percutaneous endoscopic gastrojejunostomy JET-jejunal extension tube; M—males; *n*—number; N-PEG—nutritional PEG; PEG—percutaneous endoscopic gastrostomy, PPI—proton pump inhibitors.

This single-center retrospective observational study was conducted at the 1st Department of Internal Medicine at Pavol Jozef Šafárik University and Louis Pasteur University Hospital in Košice, Slovakia, and was approved by the institutional ethics committee 2022/EK/05042. The study flow diagram is depicted in Figure 1.

Subjects who had either N-PEG or JET-PEG inserted between 1 January 2014 and 30 September 2021 were included in the study. Informed consent was obtained from all the patients or, if they were unable to provide it, from their next of kin, who were court-appointed to give consent for non-acute medical procedures. Critically ill patients in ICU have had nutritional PEG inserted in an emergency mode without prior consent as part of routine clinical care—in the case of polytrauma, unconsciousness, or ulcerative esophagitis, where the insertion of a nasogastric tube is risky. All PD patients signed a consent for the insertion of JET-PEG. Baseline patients’ characteristics and laboratory parameters were obtained from hospital medical records. Levels of hemoglobin (Hgb), C-reactive protein (CRP), leukocytes (WBC), and thrombocytes (TRC) were documented at the time of PEG insertion and subsequently three to five days after the procedure. PEG-related complications were divided into three groups: early periprocedural, late, and technical ones. Early complications were within 7 days and late complications were more than 7 days after endoscopy (Table 1) [8]. Complications were defined as minor (AGREE classification I, II) and major (AGREE IIIa, IV, V), as previously described [9]. The number of post-insertion endoscopies, the need for PEG exchange, the duration of LCIG treatment, and the person taking care of PEG (patient, family member, caregiver, health care professional) were recorded.

### 2.2. PEG Insertion Procedure

All N-PEGs were inserted by a single gastroenterologist at the Endoscopic unit using the pull technique with a video gastroscope (Gastroscope GIF-Q165or GIF-Q180, Olympus, Tokyo, Japan). All N-PEG patients had antibiotic treatment and proton pump inhibitors (PPI) at the time of insertion due to their underlying condition, as part of complex intensive care. Clear water was administered three hours after N-PEG insertion and parenteral nutrition was commenced on day 1. On day 2, enteral nutrition was initiated either in bolus or continuous pattern.

Subjects with advanced PD with severe motor fluctuations despite optimized medical therapy were considered for therapeutic JET-PEG insertion. Initially, the response to LCIG was tested by drug administration via nasojejunal tube (NJ 9 FR AbbVie, North Chicago, IL, USA) during a 3–4-day trial at the Department of Neurology UPJS and UNLP, Košice. Following a successful LCIG response test, patients were scheduled for JET-PEG insertion typically 4–6 weeks after approval from the health insurance provider was obtained. Exclusion criteria for JET-PEG insertion were patients’ disapproval, uncontrolled psychosis, comorbidities precluding gastroscopy or JET-PEG insertion, noncooperation, or noncompliance to LCIG therapy during the initial treatment trial. PEG and jejunal (J-) tubes were inserted in a single procedure under total intravenous anesthesia with continuous infusion of 1% Propofol (Fresenius Kabi, Bad Homburg, Germany) with patients in a supine or left-lateral position using gastroscope Olympus GIF-Q 165 or 180 and fluoroscopy guidance as previously described [10]. A subset of patients was in the left-lateral position during jejunal tube implantation for better manipulation and placement of tube in the duodenum. JET-PEG was then connected to an LCIG portable pump (CADD-Legacy^®^ Duodopa^®^ 1400, St. Paul, MN, USA). Prophylactic intravenous antibiotic treatment (Amoxicillin-clavulanate 1.2 g or, in subjects allergic to penicillin, ciprofloxacin 200 mg) was administered. We administered 40 mg of venous pantoprazole periprocedurally (first two days) to reduce the leak of gastric contents into the peritoneal cavity through the still immature gastrostomy canal. In the event of a granuloma onset or secretion (late complications), we resumed PPI treatment orally. Pantoprazol decreases gastric acid output and volume and increases pH within 1 h of dosing. Effects are sustained for up to 12 h following single-dose administration [8,11]. On the procedure day, patients stayed in a non-per os regime to minimize leakage of gastric content since they were fully mobile and occasionally hyperkinetic due to the presence of levodopa-induced dyskinesia.

In patients who developed late medical or technical complications associated with N-PEG or JET-PEG procedures, a revisory gastroscopy was performed. Indications included obstruction of the jejunal tube, severe abdominal or gastric pain, vomiting, and tube malposition.

### 2.3. Statistics

JASP version 0.14.1 (JASP Team, University of Amsterdam, Amsterdam, The Netherlands, 2020) was used for statistical analysis. Categorical variables were summarized as frequency counts and percentages. Continuous variables were reported as mean ± standard deviation (SD) or median and range, depending upon the normality of the population. The Shapiro–Wilk test has been used to test normality and the Kolmogorov–Smirnov test has been used to evaluate the correspondence of each parameter with a normal or non-normal distribution. Differences between categorical variables were assessed using the Pearson Chi-square test or Fisher’s exact test as appropriate. For normally distributed variables, the Student’s *t*-test or Welch´s were used to compare means between groups. Logistic regression analysis using stepwise method was used to analyze the relationship between early, late, and technical complications (response variables) and independent predictor variables. Follow-up and survival were taken in months from the date of PEG insertion until the last follow-up (September 2021) or death, whichever occurred first. Subjects with missing values as well as those with extreme values (extreme outliers) were excluded from the statistical analysis of parameters with missing/extreme values. A *p* value of ≤0.05 was considered statistically significant for all tests.

## 3. Results

### 3.1. Patient Characteristics

The present study included 87 subjects; 47 (54%; 21 [44.7%] males and 26 [55.3%] females) PD patients requiring LCIG therapy (JET-PEG group) and 40 (46%; 26 [65.0%] males and 14 [35.0%] females) subjects who needed PEG insertion due to various conditions for adequate nutrition (N-PEG group; Table 2). The most frequent indications for N-PEG insertion included neurological diseases (65%), followed by malnutrition in individuals with preserved intestinal function (17.5%), head and neck tumors (12.5%), and polytrauma (5%).

The time of PEG use was significantly longer in JET-PEG subjects (median 30, range 0.6–126 months) compared to those with N-PEG (median 5.5, range 0.11–204 months). A higher number of subjects died in the N-PEG group compared to the JET-PEG; however, all but two of those deaths were not associated with PEG insertion. The WBC counts (median 8.09 vs. 7.52 × 10^9^, *p* < 0.05), and CRP levels before PEG implantation (median 28.5 vs. 2.23 mg/L, *p* < 0.001) were significantly higher in the N-PEG group. One patient in the N-PEG group had a CRP of 653.5 mg/L after PEG insertion without other troubles. Due to the extreme outlier value of CRP, it was excluded from the statistical analysis. The subject had a Grawitz tumor with peritoneal dissemination. There was no significant difference between WBC counts and CRP levels after PEG insertion between the studied groups (median 8.15 vs. 8.02 × 10^9^, *p* > 0.05, and 45 vs. 31.55 mg/L, *p* > 0.05, respectively). However, there was a substantial rise in both parameters within the groups (*p* < 0.001 in each group). Detailed subject characteristics are summarized in Table 2.

### 3.2. Complications Associated with PEG

The occurrence of early and technical complications was significantly more frequent in JET-PEG than in N-PEG subjects, Table 2 and Table 3.

Early PEG insertion complications were noted in 28/40 (70.0%) JET-PEG subjects and 4/40 (10%) N-PEG subjects, *p* < 0.001. The most common early complications in the JET-PEG group included pain in 23 (57.5%) subjects, pneumoperitoneum in 4 (10%) subjects, and wound infection in 3 (7.5%) subjects. In N-PEG patients, pain and wound infection were present in 2 (5%) probands each, and pneumoperitoneum developed in 1 (2.5%) proband.

Late complications developed in 15/46 (32.6%) subjects from the JET-PEG group and 7/40 (17.5%) N-PEG subjects, *p* = 0.11, Table 3. The most common late complication in JET-PEG patients was infection (N = 11; 23.9%), followed by granuloma (*n* = 10; 21.7%), secretion (*n* = 7; 15.2%), pain (*n* = 2; 4.3%), migration of the tube (*n* = 1; 2.2%), and J-tube twisting (*n* = 1; 2.2%). In the group of *n*-PEG patients, late complications included infection (*n* = 2; 5%), leaking (*n* = 2; 5%), buried bumper syndrome (*n* = 2; 5%), granuloma (*n* = 1; 2.5%), and migration of the tube (*n* = 1; 2.5%).

Eighteen out of 33 (54.5%) JET-PEG probands suffered from technical complications associated with PEG compared to only 2/39 (5.1%) N-PEG patients, *p* < 0.001, Table 3. Perforation of the tube, obturation, and removal of the tube by a patient were each noted in 7 (21.2%) patients, J-tube twisting was present in 5 (15.1%), bezoars in 5 (15.1%), extraction of the tube in 3 (9.1%), and migration of the tube in 2 (6.1%) JET-PEG subjects. In the N-PEG group obstruction of the tube occurred in 2 (5.1%) subjects.

Furthermore, all three types of complications seemed to be significantly more frequent in subjects who handled the care of PEG by themselves and/or it was handled by a family member in contrast to those in whom the PEG was handled by a healthcare professional (Table 4, *p* < 0.05), however, this was not confirmed in logistic regression analysis (Table 3). The position of subjects during JET-PEG insertion (supine vs. left flank position) did not significantly affect the number of early, late, and technical complications in the JET-PEG group; Table 4).

We also assessed if there was an association between the presence of psychiatric symptoms and the number of complications. A significantly higher occurrence of early complications was found in subjects with psychiatric disorders (78.6% vs. 31.8%, *p* < 0.001, Table 3) when a direct comparison between the groups was performed. Logistic regression analysis did not find the relationship between complication rate and psychiatric disorder. Probands who did receive prophylactic PPI treatment experienced a significantly lower number of technical (11.8% vs. 41.7%, *p* = 0.005) and late (15.0% vs. 34.2%, *p* = 0.046) complications, Table 4, and similar results were obtained by logistic regression analysis for late complications (Table 3).

In probands with late and technical complications, we observed a slightly lower body mass index (BMI) than in those without complications (*p* = 0.046). On the contrary, BMI was higher in probands with early complications (*p* = 0.024, Table 5).

Subjects with JET-PEG required a higher number of endoscopies after PEG insertion than N-PEG patients (53 vs. 17 endoscopies, in 24 [51.1%] vs. 14 [35%] patients, *p* = 0.044) Table 2). When looking at the possible contributing factors we observed that re-endoscopies were more frequent in subjects who cared for PEG by themselves or this care was provided by a family member (84.2% vs. 54.2%, *p* < 0.05); were positioned on the left flank during PEG insertion (71.43% vs. 28.57%, *p* < 0.01); and also in subjects with psychiatric disorders (71.43% vs. 28.57%, *p* = 0.045, Table 6).

A lower number of endoscopies was associated with PPI prophylaxis before the PEG insertion (*p* < 0.007) and with PEG care delivered by a healthcare professional (*p* < 0.013, Table 6). In total, 70 re-endoscopies were performed after PEG insertion in 38 subjects. The reasons for re-endoscopy after PEG insertion are listed in Table 7. Ten J-tube and nine PEG replacements were performed in JET-PEG individuals and N-PEG was replaced in two subjects.

## 4. Discussion

Apart from feeding, PEG may also be used as part of a curative approach in individuals with severe PD and motor fluctuations, where dopamine precursors (levodopa–carbidopa gel) are administered through a jejunal extension tube directly to the jejunum—JET-PEG. Several previous studies report a relatively high frequency of complications related to JET-PEG, with incidence of complications up to 76%, and 17% of them reported as serious. Most commonly, these complications occurred during PEG insertion (41%) and abdominal pain was noted in 36% of patients [10,12]. Here we present the first study to directly compare the incidence and characteristics of early-, late-onset, and technical complications in subjects with N-PEG compared to JET-PEG.

In our study, early and technical complications were significantly more common among JET-PEG patients with advanced PD (70% JET-PEG vs. 10% of N-PEG). One JET-PEG patient experienced extensive pneumoperitoneum; in her case, PEG insertion led to a partial rupture of the stomach at the site of PEG insertion and required surgical intervention with PEG withdrawal and suture of the perforated stomach. Her BMI at the time of PEG insertion was 18, therefore severe malnutrition could be a precipitating factor for such an adverse event. Intestinal or stomach lacerations occur rarely with 0.5–1.3% incidence among complications associated with PEG. Pih GY et al. [13] mentioned developing of pneumoperitoneum in 9.5% within the first week of PEG placement. A small portion of patients had peritoneal irritation. All patients showed improvement with conservative management [13]. Results of the retrospective multicenter study found that age, diabetes, heart failure, higher CRP, and lower BMI all impact the risk of adverse outcomes (mortality and complications within 30 days) of PEG patients [5].

In the literature, the cause of pneumoperitoneum in PEG patients is thought to be air leakage during endoscopic insufflation of the stomach, and is a quite common and generally benign complication occurring in 4.7–57% of all cases [14,15] within three hours after PEG tube placement. Late pneumoperitoneum, three weeks after N-PEG insertion has been rarely reported [16,17,18].

Compared to previous reports that have individually assessed JET-PEG [19,20,21,22] or N-PEG [23,24] cohorts, we have also observed similar rates of late complications including stoma granulomas, infection, leaking, and painful purulent secretion. In our study, the incidence of technical complications related to the PEG and jejunal tube was high in the JET-PEG group including J-tube malfunction (caused by accidental cutting or extraction), obstruction and leaking of the tube, and tube kinging or dislocation. These complications do not occur in the N-PEG group because patients have only a gastric tube, with technical complications only 5.1%

The most common complication among our JET-PEG group was pain requiring repeated analgesic administration. All subjects with JET-PEG were fully conscious and mobile during PEG insertion compared to N-PEG individuals where only 20% were fully mobile. We suppose that hyperkinetic syndrome, especially the presence of levodopa-induced dyskinesia, in conscious PD individuals increases the painfulness due to hypermobility of the PEG catheter in the stoma with local peritoneal irritation for a few days after PEG insertion until full maturation of the gastrostomy canal Another cause of the increased prevalence of early pain in conscious PD patients may be related to noncompliance to diet regimens, e.g., eating an orange or other solid food despite instructions on the day of JET-PEG placement, which were repeatedly observed mostly in subjects with PD and cognitive decline. Similar to JET-PEG, pain was previously reported as the most common complication after PEG insertion for nutritional purposes, with an incidence of 9.2% during the first 30 days and 14.4 in the first year [24]. According to Epstein et al. [25]. procedural and abdominal pain are not commonly noted in the gastrointestinal literature because these events are expected as a consequence of the endoscopic procedure, which requires percutaneous abdominal wall puncture for PEG-J placement. In one study, rates of abdominal pain were 13% at 2 weeks post-procedure and 4% at 8 weeks [26].

In terms of the occurrence of complications depending on PEG handling, all three types of complications seemed to be more frequent in subjects who cared for PEG by themselves and/or the PEG was cared for by a family member in contrast to those in whom the PEG was handled by a healthcare professional, although this association was not confirmed in logistic regression analysis. However, proper PEG handling is vital for its functioning and prevention of complications. The position of patients during PEG insertion (supine vs. left flank position) affected the number of early and technical complications. Changing the supine position to the left flank after PEG insertion is due to better positioning of the jejunal to the deep duodenum. We hypothesize that positioning the patient with a freshly punctured stomach wall may cause leakage of gastric fluid and insufflated air into the peritoneum, with a higher risk of complications.

The most common medical complications in JET-PEG individuals were infection and granuloma. Interestingly, in a significant proportion of subjects presenting with these complications, we have observed that the external binder was not kept 1 cm away from the abdominal skin, as instructed during initial pump training, but was rather loose or cut off completely by the patient, allowing the tube to move extensively within the abdominal wall. Thus, adequate training and retraining seem to be of high priority to prevent these kinds of complications in JET-PEG patients. In our cohort, we did not observe buried bumper syndrome in JET-PEG individuals, which can be explained by a frequent manipulation of the stoma catheter in combination with regular tube rotation that may prevent mucosal overgrowth and prevent buried bumper syndrome.

WBC and CRP levels at the time of PEG insertion were significantly higher in the N-PEG group compared to the JET-PEG group N-PEG subjects were commonly intensive care unit patients with severe comorbidities including malignancies that led to the significant difference in the levels of inflammatory markers. In individuals with increasing CRP levels, abdominalgia was present but the clinical presentation of peritonitis was not observed. CRP elevation after PEG insertion is an expected phenomenon, because perforation of the stomach is followed by minimal gastric leaking. In the literature, the procedural incidence of peritonitis was 0.5–1.5% [25,27]. Peritonitis immediately after the procedure usually indicates damage to the viscus or leakage of gastric contents into the peritoneum. Peritonitis, diagnosed clinically, is not based on the presence of abnormal bacterial cultures, and may be due to early post-operative “in-and-out” movement of the PEG J-tube [25]. In our PD cohort, individuals undergoing JET-PEG insertion are fully mobile shortly after the procedure, which promotes minimal leakage of air and gastric juices with subsequent peritoneal irritation and CRP elevation. One female with JET-PEG experienced diffuse peritonitis despite antibiotic prophylaxis, and her CRP reached 289.2 mg/l. In this case, subsequent antibiotic therapy was effective, and her PEG could be preserved. This complication was probably related to malnutrition and hypoalbuminemia, which are well-recognized risk factors for higher complications and death rates after the PEG procedure [28,29,30]. CRP level is also a risk factor for predicting overall and early mortality. Using the value of 35.9 mg/dL as a cut-off showed high sensitivity in identifying patients with worse prognosis, mainly in the very early period [31,32]. CRP elevation after the PEG procedure is a consequence of peritoneal reaction to pneumoperitoneum or gastric leakage. In our opinion, it can be prevented by tighter pexy of the external bumper for the first few days after PEG insertion in mobile patients. Antibiotic prophylaxis effectively reduces the risk of early complications; patients without it experience significantly more side effects. A meta-analysis of Lipp et al. [33] showed that antibiotic prophylaxis significantly reduces the risk of infectious complications, leading to a decrease from 24.2% to 8.4%.

We observed, that patients without prophylactic PPI treatment experienced a significantly higher number of technical and late complications. We explain this observation by a lower leak of gastric juice through the stoma canal. Data assessing prophylactic PPI administration for a short-term decrease of hydrochloric acid secretion are lacking. On the other hand, several studies confirmed that long-term PPI treatment is associated with a higher complication rate when compared to non-PPI users. These complications include bowel perforation, post-PEG gastrointestinal bleeding, peritonitis, fever, pneumonia, peristomal leaks, and infection [34,35]. However, PPIs and histamine receptor blockers can be used to reduce gastric juice leakage when post-PEG bleeding is observed, or if ischemia and tissue ulcers occur due to internal bumps in the PEG gastrostomy tube [8]. Proton pump inhibitors should be initiated to minimize gastric secretion in granuloma or peristomal infection [36].

Individuals undergoing JET-PEG insertion have a higher incidence of technical complications, mainly those related to the J-tube. Compared to the N-PEG group, they underwent more re-endoscopies and analgosedations. Moreover, severe worsening of Parkinsonian motor symptoms, with the need for temporary use of rescue oral levodopa/carbidopa until the technical problem is solved, may occur due to obstruction of the J-tube. JET-PEG in individuals with PD increases their gastrointestinal morbidity, mainly with stoma pathologies. Our data show similar results as previously reported in JET-PEG individuals [19,20].

Subjects with PD treated by LCIG experience higher rates of gastroscopic procedures related to PEG insertion and treatment of complications related to JET-PEG such as a leak, granuloma, pain, and others. We showed that JET-PEG individuals underwent repeated endoscopies more frequently than N-PEG, and they needed PEG replacement due to technical complications more often. Based on our experience, the main reason for an increased number of re-endoscopies in the JET-PEG group was specifically related to issues linked with jejunal tube such as knotting, bezoar, removing, etc. Numerous tube and stoma complications related to LCIG jejunal therapy were reported. Similar to our experience, there was more accidental tube removal in patients with cognitive decline [20]. Several technical problems and complications increase the annual admission rate and contact with the hospital [37]. Inner tube complications were mostly accidental removal, kinking, or dislocation of the tube occurring during the LCIG treatment in physically active patients, sometimes suffering from disorientation [37,38]. Udd et al. [20] showed that 13 out of 60 patients in their cohort had a total of 27 tube occlusions, and eight of them (61%) had altogether ten knots in the inner tube. The intestinal tube has an angled, C-shaped tip, and this may predispose the tube to knots. Removal of the inner tube occurs more often in patients with dementia as supported by previous findings [39]. Patients with JET-PEG require a much higher degree of follow-up by gastroenterologists. Several other studies confirmed that JET-PEG individuals experience higher rates of repeated endoscopies. Sücüllü Karadağ et al. [40] published a 36.4% incidence of technical complications, half of them requiring repeated gastroscopies [41,42]. Blaise et al. [43] reported the need for repeated endoscopies in up to 68% of patients.

### Limitations and Future Perspectives

Our study has several limitations inherent to the design. This is a single center, retrospective study with a relatively small sample size. Also, ECOG status was not available, due to the retrospective nature of the study. While no PD patients with N-PEG were available for analysis, a direct comparison of endoscopic complications between JET-PEG and N-PEG patients in PD specifically was not possible and this should be analyzed in future reports. In addition, future studies should focus in more details on PD characteristics (incl. motor and non-motor symptoms and fluctuations) and neurological PD periprocedural and long-term management to understand factors that might even further reduce the occurrence of endoscopic complications.

## 5. Conclusions

Individuals with JET-PEG require the care of a multidisciplinary team consisting of a dedicated gastroenterologist, neurologist, and trained specialist nurse. Minor complications related to JET-PEG are significantly more common compared to N-PEG; however, severe complications are seen rarely. A more careful approach is specifically advised in patients presenting with low BMI and malnutrition, to prevent the risk of severe complications related to PEG placement such as gastric wall rupture or peritonitis. Periprocedural complications such as pneumoperitoneum and gastric juice leakage with peritoneum irritation can be minimized by inserting the PEG only in the supine position, with minimal movements. Also, a firmly fixed outer PEG button can reduce leakage during the first days of maturation of the stoma canal. Finally, adequate education and re-education is crucial to reduce the rates of preventable complications related especially to PEG manipulation. Overall, we can state that JET-PEG individuals more often require specialized gastroenterologist care, including re-endoscopies, compared to patients with N-PEG.

## Figures and Tables

**Table 1:** Classification of PEG-related complications.

Early (Periprocedural) Complications (Within 7 Days)	Late-Onset Medical Complications (>7 Days, after the PEG Canal Mature)	Technical Complications
Abdominal pain	Pain	Gastric tube perforation
Pneumoperitoneum	Infection	Tube kinking
Ileus	Granuloma	Bezoar
Vomiting	Peristomal leakage	Jejunal tube blockage
Peristomal wound infection	Buried bumper syndrome	Tube extraction
Fever		Tube migration
CRP elevation		
Peritonitis		

Abbreviations: CRP—C-reactive protein; PEG—percutaneous endoscopic gastrostomy.

**Table 2:** Comparison of selected parameters between studied groups of patients.

	JET-PEG	Nutrition PEG	*p* Value
Males	21 (44.7%)	26 (65.0%)	0.084
Females	26 (55.3%)	14 (35.0%)	0.084
Age at PEG insertion (years) *	66 (49–79)	63 (9–82)	0.009
PEG duration (months) *	30 (0.6–126)	5.5 (0.11–204)	<0.001
BMI (kg/m^2^) at PEG insertion *	26.08 (15.62–41.09)	23.5 (15–32)	<0.001
Hgb (g/L) at insertion *	13.35 (8.8–15.9)	11.45 (8.8–16.2)	<0.001
TRC (×10^9^) at insertion *	233.5 (114–424)	246.5 (35–498)	0.20
WBC (×10^9^) at insertion *	7.52 (3.4–18.1)	8.09 (3.2–22.4)	0.049
CRP (mg/L) at insertion *	2.23 (0.29–50.4)	28.5 (0.28–250)	<0.001
WBC (×10^9^) after insertion *	8.02 (4.07–14.05)	8.15 (3.4–31.4)	0.66
CRP (mg/L) after insertion *	31.55 (0.1–205.4)	45 (2.34–160)	0.43
ATB prophylaxis (pt *n*) †	22 (46.81%)	41 (68.33%)	0.08
ATB after PEG insertion (days) *	5 (0–14)	7 (0–30)	0.30
Early complications (pt *n*) †	28/40 (70.0%)	4/40 (10.0%)	<0.001
Technical complications (pt *n*) †	18/33 (54.5%)	2/39 (5.1%)	<0.001
Late complications (pt *n*) †	15/46 (32.6%)	7/40 (17.5%)	0.149
Left flank during PEG insertion (pt *n*) †	23/42 (54.8%)	0/40	<0.001
EGD after PEG (pt *n*) †	24/47 (51.1%)	14/40 (35.0%)	0.05
Death (pt *n*) †	18/43 (41.9%)	25/40 (62.5%)	0.06
ATB prophylaxis (pt *n*) †	22/43 (51.2%)	25/40 (62.5%)	0.377
PPI prophylaxis (pt *n*) †	19/42 (45.2%)	22/40 (55.0%)	0.508
PEG care—patient + family (pt *n*) †	37/38 (97.4%)	23/40 (57.5%)	<0.001
PEG care—healthcare professional (pt *n*) †	1/38 (2.6%)	17/40 (42.5%)	<0.001
N-PEG indications			
Neurological disease †	-	26/40 (65%)	n/a
Malnutrition †	-	7/40 (17.5%)	n/a
Head and neck tumor †	-	5/40 (12.5%)	n/a
Polytrauma †	-	2/40 (5%)	n/a

* median (range), † count/total (%). Abbreviations: ATB—antibiotics; BMI—body mass index; CRP—C-reactive protein; EGD—esophagogastroduodenoscopy; Hgb—hemoglobin; N-PEG—nutritional PEG; pt *n*—number of patients; PEG—percutaneous endoscopic gastrostomy; PPI—proton pump inhibitors; TRC—thrombocytes; WBC—white blood cells. n/a—not applicable.

**Table 3:** Logistic regression analysis of the relationship between complications and various variables.

Predictor	Odds Ratio	Confidence Interval (95%)	*p* Value
Early complications			
JET-PEG	79.79	4.93–1290.51	0.002
CRP at insertion	1.00	0.98–1.02	0.89
BMI at 1st insertion	0.94	0.77–1.15	0.55
ATB prophylaxis	0.98	0.12–8.06	0.98
PPI prophylaxis	0.34	0.04–2.96	0.33
Care of PEG by patient/family	0.23	0.02–2.70	0.24
Psychiatric disease	5.060 × 10^7^	0.00–ꝏ	0.99
Late complications			
JET-PEG	3.78	0.48–29.64	0.21
CRP at insertion	1.00	0.97–1.02	0.78
BMI at 1st insertion	0.73	0.58–0.93	0.009
ATB prophylaxis	0.88	0.10–8.15	0.90
PPI prophylaxis	0.10	0.01–0.83	0.03
Care of PEG by patient/family	1.745 × 10^8^	0.00–ꝏ	1.00
Psychiatric disease	0.52	0.4–6.17	0.61
Technical complications			
JET-PEG	27.69	1.19–643.54	0.04
CRP at insertion	1.02	0.99–1.05	0.26
BMI at 1st insertion	1.05	0.80–1.37	0.74
ATB prophylaxis	0.86	0.05–13.83	0.91
PPI prophylaxis	0.06	0.003–1.15	0.06
Care of PEG by patient/family	3.926 × 10^7^	0.00–ꝏ	1.00
Psychiatric disease	4.84	0.20–119.83	0.34

Abbreviations: ATB—antibiotics; BMI—body mass index; CRP—C-reactive protein; PEG—percutaneous endoscopic gastrostomy; PPI—proton pump inhibitors. JET—jejunal extension tube.

**Table 4:** Comparison of complications in association with various variables.

Variable			
PEG care	Patient and/or family (*n* = 60)	Nurse (*n* = 18)	
Early complications †	25/56 (44.64%)	3/18 (16.67%)	0.033
Technical complications †	19/49 (38.78%)	0/18 (0%)	0.002
Late complications †	19/59 (32.2%)	0/18 (0%)	0.006
ATB prophylaxis	ATB (*n* = 47)	No ATB (*n* = 36)	
Early complications †	14/45 (31.11%)	18/34 (52.94%)	0.050
Technical complications †	8/39 (20.51%)	11/32 (34.38%)	0.189
Late complications †	11/46 (23.91%)	9/36 (25.0%)	0.909
PPI prophylaxis	PPI (*n* = 41)	No PPI (*n* = 41)	
Early complications †	13/41 (31.71%)	18/37 (48.65%)	0.127
Technical complications †	4/34 (11.77%)	15/36 (41.67%)	0.005
Late complications †	6/40 (15.0%)	14/41 (34.15%)	0.046
Psychiatric disorder	Psychiatric disorder (*n* = 17)	No psychiatric disorder (*n* = 70)	
Early complications †	11/14 (78.57%)	21/66 (31.82%)	0.001
Technical complications †	5/10 (50.0%)	15/62 (24.19%)	0.091
Late complications †	5/17 (29.41%)	17/69 (24.64%)	0.686
Position during PEG insertion—JET-PEG group	Left flank (*n* = 23)	Supine (*n* = 19)	
Early complications †	15/21 (71.43%)	12/17 (70.59%)	0.96
Technical complications †	12/18 (66.67%)	5/14 (35.71%)	0.21
Late complications †	9/23 (39.13%)	4/19 (21.05%)	0.082
Position during PEG insertion	Left flank (*n* = 23)	Supine (*n* = 59)	
Early complications †	15/21 (71.43%)	16/57 (18.07%)	<0.001
Technical complications †	12/18 (66.67%)	7/53 (13.21%)	<0.001
Late complications †	9/23 (39.13%)	11/59 (18.64%)	0.052

† count/total (%). Abbreviations: ATB—antibiotics; *n*—number of patients; PEG—percutaneous endoscopic gastrostomy; PPI—proton pump inhibitors; JET—jejunal extension tube.

**Table 5:** Complications in association with BMI at the time of PEG insertion.

	Complications	No Complications	*p* Value
Early complications	*n* = 32	*n* = 48	
BMI (kg/m^2^)	25.79 ± 5.64	23.14 ± 4.35	0.024
Technical complications	*n* = 20	*n* = 52	
BMI (kg/m^2^)	22.47 ± 4.68	24.94 ± 5.08	0.046
Late complications	*n* = 22	*n* = 64	
BMI (kg/m^2^)	22.47 ± 4.68	24.94 ± 5.08	0.046

Abbreviations: BMI—body mass index; *n*—number.

**Table 6:** The need for EGD after PEG insertion in association with different variables.

	EGD after PEG (*n* = 38)	No EGD after PEG (*n* = 44)	*p* Value
PEG care—nurse	3/18 (16.67%)	15/18 (83.33%)	0.013
Psychiatric disorders	10/14 (71.43%)	4/14 (28.57%)	0.045
Left side during PEG insertion	15/21 (71.43%)	6/21 (28.57%)	0.010
PPI prophylaxis	12/40 (30.0%)	28/40 (70.0%)	0.007
ATB prophylaxis	18/46 (39.13%)	28/46 (60.87%)	0.187

Abbreviations: ATB—antibiotics; EGD—esophagogastroduodenoscopy; PEG—percutaneous endoscopic gastrostomy; PPI—proton pump inhibitors.

**Table 7:** Number and reasons for endoscopies after JET-PEG insertion.

Reason	Number
J-tube malfunction (accidental cutting or malposition)	15
Tube kinking (accidental)	5
Tube kinking (traumatic, with abdominal hematoma)	1
Endoscopic untangling	4
G-tube malfunction	4
Granuloma removal	2
Gastric polypectomy	1
J-tube extraction	9
Endoscopic check-up	4
Local secretion, infection, or pain	6
Gastric perforation	1
Gastroparesis	1
Total	53

## Data Availability

Laura Gombošová and Matej Škorvánek had full access to all the data in the study and takes responsibility for the integrity of the data and the accuracy of the data analysis. Data can be obtained from the corresponding author.
